# Cross-cultural adaptation to Brazilian Portuguese of the Vocal Congruence Scale and Transgender Congruence Scale

**DOI:** 10.1590/2317-1782/20232023050en

**Published:** 2023-10-30

**Authors:** Roxane de Alencar Irineu, Vanessa Veis Ribeiro, Thaís Fernandes Sebastião, Karen Crow, Miriam van Mersbergen, Mara Behlau

**Affiliations:** 1 Universidade Federal de Sergipe - UFS - Lagarto (SE), Brasil.; 2 Universidade de Brasília - UNB - Brasília (DF), Brasil.; 3 Centro de Estudos da Voz - CEV - São Paulo (SP), Brasil.; 4 School of Communication Sciences and Disorders, The University of Memphis, Memphis (TN), United States of America.; 5 Universidade Federal de São Paulo - UNIFESP - São Paulo (SP), Brasil.

**Keywords:** Transgender People, Gender Studies, Voice, Surveys and Questionnaires, Translation Process

## Abstract

**Purpose:**

to carry out the cross-cultural adaptation of the Vocal Congruence Scale (VCS) and the Transgender Scale Congruence (TSC) instruments into Brazilian Portuguese.

**Methods:**

the study was developed in two stages: cross-cultural adaptation and pre-test. 1. Cross-cultural adaptation: it was composed of a team of two speech therapists and two non-speech therapists, being responsible for the translation of the instruments into Portuguese (a speech therapist and a non-speech therapist native to Brazilian Portuguese - BP and English speakers, independently, with subsequent consensus achieved by the researchers; back-translation of the instruments into English (a speech therapist and a non-speech therapist who are native speakers of English and speakers of BP); analysis of the final version by a committee (a translator, a methodologist, and three speech therapists). Data were analyzed descriptively and inferentially.

**Results:**

In the cross-cultural adaptation process of the VCS there were adjustments in five items of the questionnaire, four of them in terms of form and one in terms of content. necessary adjustments regarding form in five items. In the pre-test, for all VCS and TSC items, the non-applicable option had a significantly lower proportion than the instrument response key options (p<0.001, for all). Finally, a translated and adapted version for Brazilian Portuguese of the Vocal Congruence Scale (VCS) and the Transgender Scale Congruence (TSC) instruments was obtained.

**Conclusion:**

The VCS and TSC were transculturally adapted to Brazilian Portuguese and named as Vocal Congruence Scale and Transgender Person Congruence Scale.

## INTRODUCTION

Transgender individuals have been a challenge in vocal clinics, mainly because intervention concerning vocal and communication aspects is a relatively recent approach in the Speech-Language Pathology field; thus, there are still few studies with scientific evidence. Many transgender women and men, crossdressers, and non-binary individuals seek speech therapy to align their voices with their gender identity since the voice can reveal their transgender status. This has been identified as a risk factor for their social existence, given the gender-based violence and transphobia frequently experienced by this population^(1)^. Thus, voice-gender congruence enhances the social relationships of transgender individuals.

Studies on the transgender population have become more frequent in the last two decades, addressing topics such as gender perception^(1-3)^; vocal parameters as gender markers^(3-5)^; the speech therapy effects on gender perception^(6-8)^, and voice self-perception in transgender individuals^(9-13)^.

The voice self-perception in this population has been useful in obtaining information about how individuals understand and identify themselves with their voices, as well as the impact it has in their lives. For this purpose, the Trans Woman Voice Questionnaire - TWVQ^(14,15)^, has been developed. This questionnaire aimed at measuring perceptions and experiences related to the transgender women's voice.

One of the goals for individuals in gender transition is to transform their voice and body to become congruent with their gender identity. Little research has been conducted on how an incongruent voice can alter identity expression and the individual's overall life satisfaction. Additionally, how a voice is perceived as incongruent impacts social, emotional, and physical aspects which are still not well understood^(16)^. The Vocal Congruence Scale (VCS) was developed in the United States of America (USA) and aims to assess the extent to which an individual's voice aligns with their sense of identity^(16)^. It provides a measurement of vocal satisfaction through a questionnaire. However, this instrument is not currently available in Brazilian Portuguese.

Body transformation is one of this population's goals, hence, it has been extensively explored. Body and voice are associated aspects and important for a person's expression, regardless of gender. Strategies for body transformation exhibit considerable variation, encompassing a range of approaches such as hormone usage, industrial silicone applications, plastic and laryngeal surgeries, and other resources. These choices are influenced by personal desires and subjective factors, and the negotiation process with professionals aims to address individual needs and socio-economic conditions, necessary to access these transformative measures^(17,18)^. These modifications aim to facilitate congruence between the image and gender identity and enhance social acceptance for transgender individuals.

Understanding the degree of congruence between physical appearance and transgender individuals’ identity would offer researchers and clinicians valuable insights. This deeper understanding would enable them to better serve this population, helping to comprehend how transgender individuals perceive the role of their physical attributes in shaping their authentic social identity experiences. To measure the comfort level of transgender individuals with their identity and appearance, the Transgender Scale Congruence (TSC) was proposed and validated in the USA^(19)^. However, this protocol also does not have a Brazilian Portuguese version.

Considering the importance of understanding the gender perception congruence in transgender individuals and the lack of tools specifically designed for this purpose in Brazil, it is essential to perform the cross-cultural adaptation of these two instruments; this is an initial step to better assess this population's needs. These instruments can help in diagnosis and provide opportunities for treatments improving vocal and body congruence for transgender individuals. Furthermore, they can provide further research to obtain evidence that will support and assist this population.

Thus, the present study aims to carry out the cross-cultural adaptation of the Vocal Congruence Scale (VCS) and the Transgender Scale Congruence (TSC) instruments in Brazilian Portuguese.

## METHODS

The present study was approved by the Research Ethics Committee of the *Universidade de Taubaté* - UNITAU, under protocol number 5.292.773 and CAAE 55653922.8.0000.5501. The study followed CNS Resolution 466/12, and all participants signed the Informed Consent Form. It is a methodological study with a cross-sectional design.

The cross-cultural adaptation of the Transgender Scale Congruence and Vocal Congruence Scale questionnaires followed the Scientific Advisory Committee (SAC) of the Medical Outcomes Trust^(20)^ and Pernambuco et al.^(21)^, recommendations. The cross-cultural adaptation included the following steps: translation, synthesis, back-translation, an expert committee review, and pre-testing.

The sample size for the translation, synthesis, back-translation, and expert committee review followed the guidelines set by the Scientific Advisory Committee (SAC) of the Medical Outcomes Trust^(20)^. The sample size for the pre-testing followed the Consensus-based Standards for the selection of health Measurement INstruments (COSMIN)^(22)^ recommendations; thus, having over 30 participants.

The translation step counted with a voice specialist speech-language pathologist and a translator with no knowledge of the construct. Both were native speakers of Brazilian Portuguese (target language) and fluent in English (source language). They were instructed to individually perform the translation into Brazilian Portuguese. Subsequently, the versions were compared by the researchers, and a synthesis version was created based on the agreement between both translators. In cases where there was no consensus, the researchers chose the option that best fitted the spoken Brazilian Portuguese while maintaining the original version concept. This version was then back-translated by another voice specialist speech-language pathologist and a translator with no knowledge of the researched area, both were native speakers of English (source language) and fluent in Brazilian Portuguese (target language).

Next, a committee of five voice specialists, including one translator, one methodologist, and three speech-language pathologists, compared the back-translated version with the original version and analyzed the semantic, conceptual, idiomatic, experiential, cultural, and operational equivalence of the VCS and TSC instruments.

At the end of these steps, the cross-culturally adapted Brazilian Portuguese versions of the two instruments were obtained. The version underwent a pre-test with the target population. A total of 38 transgender individuals participated in this step.

The inclusion criteria for the pre-test were: be Brazilian, transgender, of both genders, aged between 18 and 59 years old, with or without hormone use, and with or without speech-language pathologist follow-up. The exclusion criteria were: individuals with a medical diagnosis of lower airway infection, individuals living in Brazil but not native Brazilians, Brazilians not currently living in Brazil, illiterates, and individuals who self-reported cognitive alterations that would be difficult for them to answer the study's questionnaire.

The participants were recruited through the study's promotion on social media platforms, along with the link for participation. Data collection was conducted online using the Survey Monkey platform. Participants who accessed the platform link were instructed to read and, if they agreed to participate, digitally sign the Informed Consent Form (ICF). Those who signed the ICF were granted access to the data collection materials. The data collection consisted of a sample characterization questionnaire with eligibility criteria developed by the authors, as well as the VCS and TSC cross-culturally adapted versions.

In the VCS and TSC Brazilian Portuguese versions, the response key was supplemented with the option “not applicable.” Participants were instructed to select this option when the item was not suitable for their culture or if they were unable to understand it. In such cases, the statement had to be modified until there were no difficulties in comprehension, considering both linguistic and cultural limitations.

The Binomial Test was used for the translation and cross-cultural adaptation step; a comparison was made between the proportion of “not applicable” responses and the responses obtained through the Likert scale of the instrument's original response key: 0 - strongly disagree; 1 - disagree; 2 - neutral; 3 - agree; 4 - strongly agree. The SPSS 25.0 software was used for this analysis; the significance level was set at 5%.

## RESULTS

In both the VCS and TSC translation, unanimous agreement was observed in the process of forward and back-translation for all response options.

The VCS showed discrepancy in wording between two translators for the items 1, 2, 6, and 7: item 1.“*Parecia que minha voz me pertencia”/“Parecia que minha voz pertencia a mim”*; item 2. “*Parecia que minha voz estava funcionando normalmente”/“Parecia que minha voz estava saindo como ela normalmente sai”*; item 6. “*Eu estava pensando sobre como minha voz soava”/“Eu estava pensando na maneira como minha voz saía*”; item 7. *“Eu sinto como se minha voz soasse clara”/“Eu sinto como se minha voz saísse com clareza*”. Only item 5 presented a discrepancy in content*: “Estou satisfeito(a) pela forma como minha voz soou”/“Estou satisfeito(a) com minha voz”*. The omission of the word “sounded” from the original English version changes the sentence content: “I am satisfied with how my voice sounded”.

To synthesize both translated versions, the researchers reached a consensus, which required adjustments of items 1, 2, 6, and 7, as well as changing all item tenses. In the VCS English original version, there was no supporting sentence before presenting the items, and the questionnaire was written in the past tense. After reaching a consensus, an introductory sentence was included: “*Quando eu me comunico com as pessoas*”, which also included changing the sentence to the present tense. For item 5, which presented a discrepancy regarding the content, the researchers decided to keep the term “*sounded*”: “*Fico satisfeito(a) com o som da minha voz*”.

In the VCS back-translation step, there was disagreement regarding the form of all items among the translators. Regarding the content, there was only disagreement in item 9 - “I feel that my voice accurately reflects my sense of humor” / “I feel my voice reflects my mood in a correct way”. The terms “accurately/*precisamente*” and “correct way/*maneira correta*,” although similar, have different meanings. Thus, these specific terms were omitted, as the word “reflect/*reflete*” provides the intended meaning: “*Sinto que minha voz reflete como está meu humor*”/ “I feel that my voice reflects my mood.”

After the expert committee of speech-language pathologists review, the VCS final version underwent adjustments in items 1, 4, 8, 9, and 10 (Chart 1).

**Chart 1:** Cross-cultural adaptation process of the Vocal Congruence Scale and the Transgender Scale Congruence into Brazilian Portuguese

**Original English version**	**Translation to Brazilian Portuguese**	**Back-translation into English.**	**An expert committee of speech-language pathologists/ Final translated and adapted version**
***Vocal Congruence Scale***	**Escala de Congruência Vocal**	***Vocal Congruence Scale***	**Escala de Congruência Vocal**
0. s*trongly disagree*	0.discordo totalmente	0.I totally disagree	0.discordo totalmente
1. d*isagree*	1.discordo	1.disagree	1.discordo
2. n*eutral*	2.neutro	2.neutral	2.neutro
3. a*gree*	3.concordo	3.I agree	3.concordo
4. s*trongly agree*	4.concordo totalmente	4.I totally agree	4.concordo totalmente
	Quando eu me comunico com as pessoas...	When I communicate with people…	Quando eu me comunico com as pessoas...
1*. It seemed like my voice belonged to me*	T1.Parecia que minha voz me pertencia	R1.*I feel that my voice belongs to me*	Sinto que a voz é minha
	T2.Parecia que minha voz pertencia a mim	R2.*I feel my voice belongs to me*	
	VCP.Sinto que a minha voz me pertence		
2. *It seemed like my voice was functioning as it normally does*	T1.Parecia que minha voz estava funcionando normalmente	R1.*I feel that my voice functions normally*	Sinto que minha voz funciona normalmente
	T2.Parecia que minha voz estava saindo como ela normalmente sai	R2*.I feel my voice functioning normally*	
	VCP.Sinto que minha voz funciona normalmente		
*3. It seemed like my voice reflected who I am*	T1.Parecia que minha voz refletia quem eu sou	R1*.I feel that my voice reflects who I am*	Sinto que minha voz reflete quem eu sou
	T2. Parecia que minha voz refletia quem eu sou	R2*.I feel my voice reflects who I am*	
	VCP.Sinto que minha voz reflete quem eu sou		
*4. It seemed like I was in control of my voice*	T1.Parecia que eu estava no controle da minha voz	R1.*I feel that I’m in control of my own voice*	Sinto que controlo minha voz
	T2.Parecia que eu estava no controle da minha voz	R2.*I feel control over my voice*	
	VCP.Sinto que estou no controle da minha voz		
5. *I am satisfied with how my voice sounded*	T1.Estou satisfeito(a) pela forma como minha voz soou	R1.*I feel satisfied with the sound of my voice*	Fico satisfeito(a) com o som da minha voz
	T2.Estou satisfeito(a) com minha voz	R2.*I feel satisfied with my voice*	
	VCP.Fico satisfeito(a) com o som da minha voz		
6*. I was thinking about the way my voice sounded*	T1.Eu estava pensando sobre como minha voz soava	R1.*I’ve been thinking about how my voice resonates/echo*	Fico pensando sobre como minha voz soa
	T2.Eu estava pensando na maneira como minha voz saía	R2.*keep thinking about how my voice sounds*	
	VCP.Fico pensando sobre como minha voz soa		
7. *I feel as though my voice sounded clear*	T1.Eu sinto como se minha voz soasse clara	R1*.I feel that my voice is clear*	Sinto que minha voz é clara
	T2.Eu sinto como se minha voz saísse com clareza	R2*.I feel my voice clear*	
	VCP.Sinto que minha voz é clara		
8. *I feel as though I was speaking in a way that I was easily understood*	T1.Eu sinto como se estivesse falando de uma maneira que eu fosse facilmente compreendida	R1*.I feel that I talk in a way that is easily understood by others*	Sinto que minha fala é facilmente compreendida
	T2.Eu sinto como se eu estivesse falando de uma maneira que eu fosse facilmente entendido(a)	R2.*I feel I talk in a way so that I am easily understood*	
	VCP.Sinto que falo de uma forma que sou facilmente compreendido(a)		
9. *I feel as though my voice accurately reflected my mood*	T1.Sinto como se minha voz refletisse com precisão meu humor.	R1.*I feel that my voice accurately reflects my sense of humor*	Sinto que minha voz reflete como está meu humor
	T2.Eu sinto como se minha voz refletisse com precisão meu humor	R2.*I feel my voice reflects my mood in a correct way*	
	VCP.Sinto que minha voz reflete corretamente meu humor		
10*. I feel as though my voice reflected my gender*	T1.Eu sinto como se minha voz refletisse meu gênero.	R1*.I feel that my voice reflects my gender*	Sinto que minha voz reflete meu gênero
	T2.Eu sinto como se minha voz refletisse meu gênero	R2.*I feel my voice reflects my gender*	
	VCP.Sinto que minha voz reflete meu gênero		
***Transgender Congruence Scale***	**Escala de Congruência da Pessoa Transgênero**	***Congruence scale of a transgender person***	**Escala de Congruência da Pessoa Transgênero**
*0. strongly Disagree*	0.discordo totalmente	*0.I totally disagree*	0.discordo totalmente
*1. disagree*	1.discordo	*1.disagree*	1.discordo
*2. neutral*	2.neutro	*2.neutral*	2.neutro
*3. agree*	3.concordo	*3.I agree*	3.concordo
*4. strongly Agree*	4.concordo totalmente	*4.I totally agree*	4.concordo totalmente
1*. My outward appearance represents my gender identity*	T1.Meu corpo físico representa minha identidade de gênero	R1.My physical body represents my gender identity	Minha aparência representa minha identidade de gênero
	T2.Meu corpo físico representa minha identidade de gênero	R2. My physical body represents my gender identity	
	VCP. Meu corpo físico representa minha identidade de gênero		
2*. I experience a sense of unity between my gender identity and my body.*	T1. Eu sinto uma unidade entre minha identidade de gênero e meu corpo	R1. I feel that my gender and my body connected with each other	Eu sinto que existe unidade entre meu gênero e meu corpo
	T2. Eu experimento um senso de unidade entre meu gênero e meu corpo	R2. I feel there is a unity between my gender and my body	
	VCP. Eu sinto que existe uma unidade entre meu gênero e meu corpo		
3. *My physical appearance adequately expresses my gender identity*	T1.Minha aparência física expressa adequadamente minha identidade de gênero	R1.My physical appearance adequately expresses my gender identity	Minha aparência física expressa minha identidade de gênero
	T2.Minha aparência física expressa adequadamente minha identidade de gênero	R2.My physical appearance expresses in an adequate way my gender identity	
	VCP.Minha aparência física expressa adequadamente minha identidade de gênero		
4. *I am generally comfortable with how others perceive my gender identity when they look at me*	T1.Geralmente me sinto confortável com a forma como os outros percebem minha identidade de gênero quando olham para mim	R1.I generally feel comfortable with how other people notice my gender identity and look at me	Eu me sinto confortável com a forma que os outros percebem minha identidade de gênero quando olham para mim
	T2.Eu geralmente me sinto confortável com a maneira que os outros percebem minha identidade de gênero quando olham para mim	R2.Normally I feel comfortable about how others perceive my gender identity when they look at me	
	VCP.Eu geralmente me sinto confortável como os outros percebem minha identidade de gênero ao olharem para mim		
5*. My physical body represents my gender identity*	T1.Meu corpo físico representa minha identidade de gênero	R1.My physical body represents my gender identity	Meu corpo físico representa minha identidade de gênero
	T2.Meu corpo físico representa minha identidade de gênero	R2.My physical body represents my gender identity	
	VCP. Meu corpo físico representa minha identidade de gênero		
*6. The way my body currently looks does not represent my gender identity*	T1.A aparência do meu corpo atualmente não representa minha identidade de gênero	R1.The actual appearance of my physical body is not an accurate representation of my gender identity	A aparência do meu corpo não representa minha identidade de gênero
	T2.A maneira que meu corpo atualmente aparenta não representa minha identidade de gênero	R2.My actual appearance does not represent my gender identity	
	VCP.A aparência atual do meu corpo não representa minha identidade de gênero		
7*. I am happy with the way my appearance expresses my gender identity*	T1.Estou feliz com a forma como minha aparência expressa minha identidade de gênero	R1.I am happy with the way that my appearance expresses my gender identity	Eu estou feliz com a forma que minha aparência expressa minha identidade de gênero
	T2.Eu estou feliz com a maneira que minha aparência expressa minha identidade de gênero	R2. I am happy with the way my appearance expresses my gender identity	
	VCP.Eu estou feliz com a maneira que minha aparência expressa minha identidade de gênero		
8. *I do not feel that my appearance reflects my gender identity*	T1.Não sinto que minha aparência reflita minha identidade de gênero	R1.I don’t feel that my appearance reflects my gender identity	Eu estou feliz com a forma que minha aparência expressa minha identidade de gênero
	T2.Eu não sinto que minha aparência reflete minha identidade de gênero	R2.I do not feel that my appearance reflects my gender identity	
	VCP.Eu não sinto que minha aparência reflete minha identidade de gênero		
9. *I feel that my mind and body are consistent with one another*	T1.Sinto que minha mente e meu corpo são consistentes um com o outro	R1.I feel that my body and mind are coherent with one another	Eu sinto que minha mente e corpo são consistentes um com o outro
	T2.Eu sinto que minha mente e corpo são consistentes um com o outro	R2.I feel my mind and body are coherent with one another	
	VCP.Eu sinto que minha mente e corpo são coerentes um com o outro		
10. *I am not proud of my gender identity*	T1.Eu não tenho orgulho da minha identidade de gênero	R1.I don’t feel proud of my gender identity	Eu não me orgulho da minha identidade de gênero
	T2.Eu não estou orgulhoso(a) da minha identidade de gênero	R2.I do not feel proud about my gender identity	
	VCP. Eu não me orgulho da minha identidade de gênero		
11. *I am happy that I have the gender identity that I do*	T1.Estou feliz por ter a identidade de gênero que tenho	R1.I am happy with my gender identity	Eu estou feliz com minha identidade de gênero
	T2.Eu estou feliz por ter a identidade de gênero que tenho	R2.I am happy about the gender identity I possess	
	VCP.Eu estou feliz pela identidade de gênero que tenho		
12. *I have accepted my gender identity*	T1.Aceitei minha identidade de gênero	R1.I accept my gender identity	Eu aceito minha identidade de gênero
	T2.Eu aceitei minha identidade de gênero	R2.I accept my gender identity	
	VCP.Eu aceito minha identidade de gênero		

Caption: T1 - Translator 1 English-Portuguese; T2 - Translator 2 English-Portuguese; B1 - Back-translator 1 Portuguese-English; B2 - Back-translator 2 Portuguese-English; BPV BPV - Brazilian Portuguese version by consensus of two translators.

In the TSC questionnaire, there was disagreement regarding the form during the translation step for items 2, 4, 6, 7, and 10: item 2. *“Eu sinto uma unidade entre minha identidade de gênero e meu corpo”/“Eu experimento um senso de unidade entre meu gênero e meu corpo”;* item 4. *“Geralmente me sinto confortável com a forma como os outros percebem minha identidade de gênero quando olham para mim”/“Eu geralmente me sinto confortável com a maneira que os outros percebem minha identidade de gênero quando olham para mim”;* item 6. *“A aparência do meu corpo atualmente não representa minha identidade de gênero”/“A maneira que meu corpo atualmente aparenta não representa minha identidade de gênero”;* item 7. *“Estou feliz com a forma como minha aparência expressa minha identidade de gênero”/“Eu estou feliz com a maneira que minha aparência expressa minha identidade de gênero”;* item 10. *“Eu não tenho orgulho da minha identidade de gênero”/“Eu não estou orgulhoso(a) da minha identidade de gênero”.*

In the consensus, the disagreements were resolved, and the items were simplified, resulting in synthesized versions: item 2. *“Eu sinto que existe uma unidade entre meu gênero e meu corpo”;* item 4*.“Eu geralmente me sinto confortável como os outros percebem minha identidade de gênero ao olharem para mim”,* item 6*.“A aparência atual do meu corpo não representa minha identidade de gênero”; item 7.“Eu estou feliz com a maneira que minha aparência expressa minha identidade de gênero”;* and, item 10*.“Eu não me orgulho da minha identidade de gênero”.*

During the TSC back-translation step, there were disagreements regarding the form for items 2, 3, 4, 6, and 11: item 2. “I feel that my gender and my body connected with each other”/ “I feel there is a unity between my gender and my body”; item 3. “My physical appearance adequately expresses my gender identity”/ “My physical appearance expresses in an adequate way my gender identity”; item 4. “I generally feel comfortable with how other people notice my gender identity and look at me”/ “Normally I feel comfortable about how others perceive my gender identity when they look at me”; item 6. “The actual appearance of my physical body is not an accurate representation of my gender identity”/ “My actual appearance does not represent my gender identity”; item 11. “I am happy with my gender identity”/ “I am happy about the gender identity I possess”.

After the expert committee of speech-language pathologists review, the TSC final version underwent adjustments in items 1, 3, 4, 6, 7, 8, and 9 (Chart 1).

During the pre-test step, the “not applicable” option for all items in both the VCS and the TSC had a significantly lower proportion compared to the options typically found in the instruments response key (p<0.001, for all), as indicated in Table 1.

**Table 1:** Comparison of the proportion of items with usual responses and the proportion of items with non-applicable responses in the Voice Congruence Scale and Transgender Scale Congruence

Voice Congruence Scale items	0-4		Not Applicable		p-value
	n	Observed	n	Observed	
		proportion		proportion	
Voice Congruence Scale					
1-Sinto que a voz é minha	37	0.97	1	0.03	0.000
2-Sinto que minha voz funciona normalmente	38	1.00	0	0.00	0.000
3-Sinto que minha voz reflete quem eu sou	38	1.00	0	0.00	0.000
4-Sinto que controlo minha voz	37	0.97	1	0.03	0.000
5-Fico satisfeito(a) com o som da minha voz	38	1.00	0	0.00	0.000
6-Fico pensando como minha voz soa	37	0.97	1	0.03	0.000
7-Sinto que minha voz é clara	37	0.97	1	0.03	0.000
8-Sinto que minha fala é facilmente compreendida	38	1.00	0	0.00	0.000
9-Sinto que minha voz reflete como está meu humor	38	1.00	0	0.00	0.000
10-Sinto que minha voz reflete meu gênero	38	1.00	0	0.00	0.000
Transgender Scale Congruence					
1-Minha aparência representa minha identidade de gênero	38	1.00	0	0.00	0.000
2-Eu sinto que existe unidade entre meu gênero e meu corpo	37	0.97	1	0.03	0.000
3-Minha aparência física expressa minha identidade de gênero	38	1.00	0	0.00	0.000
4-Eu me sinto confortável com a forma que os outros percebem	38	1.00	0	0.00	0.000
minha identidade de gênero quando olham para mim					
5-Meu corpo físico representa minha identidade de gênero	37	0.97	1	0.03	0.000
6-A aparência do meu corpo não representa minha identidade de	36	0.95	2	0.05	0.000
gênero					
7-Eu estou feliz com a forma que minha aparência expressa minha	38	1.00	0	0.00	0.000
identidade de gênero					
8-Eu não sinto que minha aparência reflete minha identidade de	38	1.00	0	0.00	0.000
gênero					
9-Eu sinto que minha mente e corpo são consistentes um com o	37	0.97	1	0.03	0.000
outro					
10-Eu não me orgulho da minha identidade de gênero	37	0.97	1	0.03	0.000
11-Eu estou feliz com minha identidade de gênero	36	0.95	2	0.05	0.000
12-Eu aceito minha identidade de gênero	36	0.95	2	0.05	0.000

Binomial Test

**Caption:** n = absolute frequency

## DISCUSSION

The questionnaire transcultural adaptation is the initial step for the validation process, which is essential to enable the instrument used in another language and culture^(23)^.

After the translation, synthesis, back-translation, and the expert committee review, some adjustments in the instrument’s items were necessary for a better comprehension of Brazilian culture. The VCS original English version uses the past tense; the questionnaire was produced during a study that aimed to identify the perception immediately after performing a speech task. However, the verb tense had to be adjusted to the present tense.

Before each item, an additional introductory sentence was needed in the VCS Brazilian Portuguese version: “Quando eu me comunico com as pessoas...”/ When I communicate with people”. This sentence addition contributed to the items' comprehension, enabling their seamless integration with communication.

Also, the TCS underwent item simplification, omission of terms that did not significantly contribute to the sentence's structure, and word changes.

The pre-test confirmed that all items in both instruments were applicable and comprehensible to the transgender population. In the VCS the “not applicable” option was used by one single person for items 1, 4, 6, and 7: item 1. *“Sinto que a voz é minha”; item 4. “Sinto que controlo minha voz”;* item 6. *“Fico pensando como minha voz soa”;* item 7. *“Sinto que minha voz é clara”.*

For the TSC, one person answered “not applicable” in the items 2, 5, 9 and 10: item 2. *“Eu sinto que existe unidade entre meu gênero e meu corpo”;* item 5. *“Meu corpo físico representa minha identidade de gênero”;* item 9. *“Eu sinto que minha mente e corpo são consistentes um com o outro”;* item 10. *“Eu não me orgulho da minha identidade de gênero”.* Also for the TSC, two persons answered “not applicable” for the items 6, 11 and 12: *“A aparência do meu corpo não representa minha identidade de gênero”, “Eu estou feliz com minha identidade de gênero”, “Eu aceito minha identidade de gênero”.* However, these instances were isolated, and the answers did not correspond to the responses of the entire sample. Therefore, no further adjustments were necessary.

The current study's transcultural adaptation has resulted in the development of the Brazilian Portuguese versions of both questionnaires. They are applicable and easy to understand for transgender individuals, which will contribute to a more specific and suitable speech and language assessment. The final versions will undergo the next validation process steps to confirm the number of items and factors; also, the instrument's psychometric properties will be analyzed. After this final step, the instruments will be ready for clinical and research use with the transgender population.

## CONCLUSION

The cross-cultural adaptation of the VCS and TSC questionnaires for Brazilian Portuguese involved necessary adjustments to ensure their appropriateness in the Brazilian context. The resulting versions, namely the “*Escala de Congruência Vocal*” and “*Escala de Congruência da Pessoa Transgênero,*” were found to be easily understandable and applicable in both clinical and research settings. These adapted instruments offer insights into the gender understanding, vocal and body congruence experienced by transgender individuals.
