# Supplementary material for: Cross-cultural adaptation to Brazilian Portuguese of the Vocal Congruence Scale and Transgender Congruence Scale
Source: Codas. 2023 Oct 30;36(2):e20230050. doi: 10.1590/2317-1782/20232023050en (PMC10715632; doi:10.1590/2317-1782/20232023050en)
Supplement: Translation

# Translation: Adaptação transcultural para o português Brasileiro do Vocal Congruence Scale e Transgender Congruence Scale

**DOI:** 10.1590/2317-1782/20232023050pt

**Authors:** Roxane de Alencar Irineu, Vanessa Veis Ribeiro, Thaís Fernandes Sebastião, Karen Crow, Miriam van Mersbergen, Mara Behlau

**Affiliations:** 1 Universidade Federal de Sergipe - UFS - Lagarto (SE), Brasil.; 2 Universidade de Brasília - UNB - Brasília (DF), Brasil.; 3 Centro de Estudos da Voz - CEV - São Paulo (SP), Brasil.; 4 School of Communication Sciences and Disorders, The University of Memphis, Memphis (TN), United States of America.; 5 Universidade Federal de São Paulo - UNIFESP - São Paulo (SP), Brasil.

**Keywords:** Pessoas Transgênero, Estudos de Gênero, Voz, Inquéritos e Questionários, Processo de Tradução

## Abstract

**Objetivo:**

realizar a adaptação transcultural dos instrumentos Vocal Congruence Scale (VCS) e o Transgender Scale Congruence (TSC) para o português brasileiro.

**Método:**

o estudo foi desenvolvido em duas etapas: adaptação transcultural e pré-teste. 1. Adaptação transcultural: foi composta por uma equipe de dois fonoaudiólogos e dois não-fonoaudiólogos, sendo responsáveis pela tradução para o português (um fonoaudiólogo e um não-fonoaudiólogo nativos do português brasileiro - PB e falantes do inglês), de modo independente, com posterior consenso realizado pelos pesquisadores; retrotradução para o inglês (um fonoaudiólogo e um não-fonoaudiólogo nativos do inglês e falantes do PB); análise da versão final por um comitê (um tradutor, um metodologista, e três fonoaudiólogos). 2. Etapa de pré-teste: os instrumentos foram aplicados em 38 indivíduos transgêneros (29 mulheres trans, 2 travestis e 7 homens trans), sendo acrescido na chave de resposta a opção “não aplicável”. Os dados foram analisados de forma descritiva e inferencial.

**Resultados:**

No processo de adaptação transcultural do VCS houve ajustes em cinco itens do questionário, quatro deles quanto à forma e um quanto ao conteúdo. Para o TSC também foram necessários ajustes quanto a forma em cinco itens. No pré-teste, para todos os itens dos instrumentos, a opção não aplicável apresentou proporção significativamente menor que as opções da chave de resposta dos instrumentos. Por fim, foi obtida uma versão traduzida e adaptada para o português dos dois instrumentos.

**Conclusão:**

Os instrumentos foram transculturalmente adaptados para o português brasileiro e nomeados como Escala de Congruência Vocal e Escala de Congruência da Pessoa Transgênero.

## INTRODUÇÃO

A transgeneridade tem sido um desafio na clínica vocal principalmente pelo fato de a intervenção fonoaudiológica sobre os aspectos vocais e de comunicação ser uma abordagem relativamente recente na Fonoaudiologia, com um quantitativo ainda limitado de estudos com evidência científica. Muitas mulheres e homens transgêneros, travestis e não-binários procuram o atendimento fonoaudiológico para adequar suas vozes ao gênero de sua identificação, visto que, a voz pode anunciar sua transgeneridade. Isso tem sido elencado como um fator de risco para sua existência social, em virtude da violência de gênero e transfobia sofridas rotineiramente por esta população^(1)^. Sendo assim, a congruência voz-gênero favorece as relações sociais das pessoas transgêneros.

Estudos sobre a população trans tem sido mais frequentes nas duas últimas décadas, abordando temáticas sobre a percepção de gênero^(1-3)^; parâmetros vocais marcadores de gênero^(3-5)^; efeitos da terapia fonoaudiológica na percepção de gênero^(6-8)^, e, autopercepção vocal de pessoas trans^(9-13)^.

A autopercepção vocal dessa população tem sido útil para obtenção de informações sobre como o indivíduo entende e se identifica com sua voz, e qual o impacto dela nos diversos cenários de sua vida. Com essa finalidade têm-se o *Trans Woman Voice Questionnaire - TWVQ* ^(14,15)^, um questionário que busca mensurar as percepções e experiências relacionadas à voz para mulheres trans.

Uma das metas das pessoas em transição de gênero é transformar sua voz e seu corpo para ficar congruente com o gênero de sua identificação. Pouco tem se investigado sobre como uma voz incongruente pode alterar a expressão da identidade e a própria satisfação do indivíduo com sua vida. Além disso, não se conhece de que maneira uma voz considerada incongruente por uma pessoa impacta negativamente aspectos sociais, emocionais e físicos^(16)^. O *Vocal Congruence Scale* (VCS) foi desenvolvido nos EUA e busca avaliar até que ponto a voz de um indivíduo é congruente com o senso de identidade^(16)^ e oferece, por meio de um questionário, uma mensuração da satisfação vocal. Porém, não há uma versão do instrumento em português brasileiro.

A congruência corporal vem sendo também muito explorada por ser a transformação corporal uma das metas dessa população. Corpo e voz são, portanto, aspectos associados e importantes para a expressão de uma pessoa, independentemente do gênero. As estratégias da transformação dos corpos podem variar entre uso de hormônios; aplicações de silicone industrial; realização de cirurgias plásticas e de cirurgias laríngeas, entre outros recursos cuja escolha é mediada pelas vontades pessoais e subjetividades em negociação com necessidades profissionais e condições socioeconômicas para adquiri-las^(17,18)^. Tais modificações buscam facilitar a congruência da imagem à identidade de gênero e a aceitação social da pessoa trans.

A compreensão do grau de congruência entre a aparência física e a identidade das pessoas transgêneros proporcionaria aos pesquisadores e aos clínicos que atendem essa população uma percepção da medida em que os indivíduos transgêneros percebem que seus atributos físicos ajudam ou interferem na vivência de sua identidade social, de forma autêntica. Para mensurar o conforto dos indivíduos transgêneros com a identidade e a aparência foi proposto e validado, também nos EUA, o *Transgender Scale Congruence* (TSC)^(19)^. Este protocolo também não possui versão em português brasileiro.

Considerando-se a importância de compreender a percepção de congruência de gênero nas pessoas trans, bem como o fato de que não havia ferramentas com essa finalidade para serem utilizados no Brasil, torna-se importante realizar a adaptação transcultural desses dois instrumentos, um passo inicial para avaliar melhor as necessidades dessa população. Baseado nos argumentos apresentados, entende-se que esses instrumentos poderão auxiliar no diagnóstico e, também, propiciar a oferta de tratamentos que melhorem a congruência vocal e corporal do sujeito transgênero, além da realização de pesquisas para obtenção de evidências para auxiliar essa população.

Desta forma, este estudo tem como objetivo realizar a adaptação transcultural para o português brasileiro do *Vocal Congruence Scale* e *Transgender Congruence Scale*.

## MÉTODO

O presente estudo foi aprovado pelo Comitê de Ética em Pesquisa da Universidade de Taubaté - UNITAU, sob parecer 5.292.773 e CAAE 55653922.8.0000.5501. O estudo seguiu a Resolução CNS 466/12, e todos os participantes assinaram o Termo de Consentimento Livre Esclarecido. Trata-se de um estudo de natureza metodológica e delineamento transversal.

As etapas de adaptação transcultural dos questionários *Transgender Scale Congruence* e do *Vocal Congruence Scale* seguiram as recomendações do *Scientific Advisory Committee* (*SAC*) do *Medical Outcomes Trust* ^(20)^ e de Pernambuco et al.^(21)^. A adaptação transcultural foi composta pelas etapas de tradução, síntese, retrotradução, revisão pelo comitê de especialistas e pré-teste.

O tamanho amostral para as etapas de tradução, síntese, retrotradução e revisão pelo comitê de especialista seguiu o determinado pelo *Scientific Advisory Committee* (*SAC*) do *Medical Outcomes Trus* ^(20)^. O tamanho da amostra para o pré-teste seguiu a determinação de ser acima de 30 participantes do Consensus- based Standards for the selection of health Measurement INstruments (COSMIN)^(22)^. 

Para a etapa de tradução foram convidados para participar um fonoaudiólogo especialista em voz e um tradutor sem conhecimento sobre o construto, ambos nativos do PB (idioma alvo) e falantes do inglês (idioma fonte). Eles foram instruídos a realizar, de forma individual, a tradução para o português brasileiro. Em seguida, as versões foram confrontadas pelos pesquisadores e foi elaborada uma versão de síntese com base na concordância entre os tradutores. Nos casos em que não houve concordância, os pesquisadores optaram pela escolha da opção dos revisores que melhor se adequava ao PB correntemente falado, mantendo o conceito da versão original. Essa versão foi retrotraduzida por um fonoaudiólogo especialista em voz e um tradutor sem conhecimento na área pesquisada, nativos do inglês (idioma fonte) e falantes do PB (idioma alvo).

Em seguida, um comitê formado por cinco profissionais especialistas em voz, sendo um tradutor, um metodologista, e três fonoaudiólogos, compararam a versão retrotraduzida com a original e analisaram a equivalência semântica, conceitual, idiomática, experiencial, cultural e operacional dos instrumentos *VCS e TSC.*

Ao final dessas etapas foram obtidas as versões adaptadas transculturalmente para o português brasileiro dos dois instrumentos, que foram submetidas ao pré-teste com a população-alvo. Nessa etapa foi necessário a participação de 38 pessoas trans.

Os critérios de inclusão para a participação no pré-teste foram ser brasileiro/a, transgênero/a, de ambos os gêneros, com idade entre 18 e 59 anos, com ou sem uso de hormônio, com ou sem acompanhamento fonoaudiológico. Foram excluídos deste estudo os indivíduos com diagnóstico médico de alterações de vias aéreas inferiores, indivíduos que residiam no Brasil mas não eram nativos brasileiros, brasileiros que não residiam no Brasil atualmente, analfabetos, e indivíduos com presença de déficit cognitivo autorreferido que o impedisse de responder adequadamente o que lhe foi solicitado neste estudo.

Os/as participantes foram recrutados/as por meio da divulgação do estudo em mídias sociais, juntamente com o link para participação. A coleta de dados foi realizada online pela plataforma *Survey Monkey*. Os participantes que acessaram o link da plataforma foram orientados a ler e, em caso de concordância em participar, assinarem digitalmente o Termo de Consentimento Livre e Esclarecido (TCLE). Os participantes que assinaram o TCLE tiveram acesso ao material da coleta de dados. A coleta de dados foi composta por um questionário de caracterização da amostra com os critérios de elegibilidade elaborado pelos autores, e pelas versões transculturalmente adaptadas do *VCS* e do *TSC*.

Nos instrumentos *VCS* e *TSC* em português brasileiro foi acrescida na chave de resposta a opção “não aplicável”. Os participantes foram orientados a assinalar essa opção quando o item não estivesse adequado para sua cultura, ou se não fosse possível compreendê-lo. Caso isso acontecesse, a afirmativa deveria ser modificada até que não houvesse dificuldades na compreensão, tanto por deficiências linguísticas, quanto culturais.

Para a etapa de tradução e adaptação transcultural foi utilizado o Teste Binomial, no qual foi realizada a comparação da proporção de respostas “não aplicável” e de respostas obtidas pela escala de Likert presentes na chave de resposta original do instrumento: 0 - nunca, 1 - quase nunca, 2 - às vezes, 3 - quase sempre, 4 - sempre. Utilizou-se o software SPSS 25.0 e foi estabelecido o nível de significância de 5%.

## RESULTADOS

Houve concordância na tradução e retrotradução de todas as opções da chave de resposta dos questionários *VCS* e *TSC*.

Na etapa de tradução dos itens do *VCS* houve discordância de forma entre os dois tradutores nos itens 1, 2, 6 e 7: item 1.“Parecia que minha voz me pertencia”/“Parecia que minha voz pertencia a mim”; item 2. “Parecia que minha voz estava funcionando normalmente”/“Parecia que minha voz estava saindo como ela normalmente sai”; item 6. “Eu estava pensando sobre como minha voz soava”/“Eu estava pensando na maneira como minha voz saía”; item 7. “Eu sinto como se minha voz soasse clara”/“Eu sinto como se minha voz saísse com clareza”. Houve discordância quanto ao conteúdo apenas do item 5: “Estou satisfeito(a) pela forma como minha voz soou”/“Estou satisfeito(a) com minha voz”. Entende-se que a omissão da palavra “sounded” da versão original do inglês modifica o conteúdo da frase: *“I am satisfied with how my voice sounded”*.

Para a síntese das versões traduzidas, os pesquisadores elaboraram um consenso, havendo necessidade de ajuste da forma de apresentação dos itens 1, 2, 6 e 7 e do tempo verbal de todos os itens. Na versão original em inglês do *VCS* não existia nenhuma frase de apoio antes da apresentação dos itens, e o tempo verbal do questionário estava no passado. Após o consenso foi inserida a frase inicial: “Quando eu me comunico com as pessoas”, e transformado o tempo verbal para o presente do indicativo. Para o item 5 houve discordância do conteúdo, os pesquisadores entenderam que era necessário considerar o termo “*sounded*”: “Fico satisfeito(a) com o som da minha voz”.

Na etapa da retrotradução do *VCS* houve discordância quanto a forma em todos os itens entre os tradutores. Quanto ao conteúdo, houve discordância apenas no item 9 - *“I feel that my voice accurately reflects my sense of humor”/“I feel my voice reflects my mood in a correct way”*. O termo “*accurately*/precisamente” e “*correct way*/maneira correta” ainda que semelhantes, dizem coisas diferentes. Optou-se por omitir os referidos termos, visto que a palavra “reflect/reflete” contempla a necessidade da frase: “Sinto que minha voz reflete como está meu humor”.

A versão final do *VCS* após reunião do comitê de fonoaudiólogos especialistas obteve ajustes nos itens 1, 4, 8, 9 e 10 (Quadro 1).

**Quadro 1:** Processo de adaptação transcultural do *Vocal Congruence Scale* e do *Transgender Scale Congruence* para o português brasileiro

**Itens da versão original em inglês**	**Tradução para o português brasileiro**	**Retrotradução para o inglês**	**Comitê de fonoaudiólogos especialistas/ Versão final traduzida e adaptada**
***Vocal Congruence Scale***	**Escala de Congruência Vocal**	***Vocal Congruence Scale***	**Escala de Congruência Vocal**
0. s*trongly disagree*	0.discordo totalmente	0.I totally disagree	0.discordo totalmente
1. d*isagree*	1.discordo	1.disagree	1.discordo
2. n*eutral*	2.neutro	2.neutral	2.neutro
3. a*gree*	3.concordo	3.I agree	3.concordo
4. s*trongly agree*	4.concordo totalmente	4.I totally agree	4.concordo totalmente
	Quando eu me comunico com as pessoas...	When I communicate with people…	Quando eu me comunico com as pessoas...
1*. It seemed like my voice belonged to me*	T1.Parecia que minha voz me pertencia	R1.*I feel that my voice belongs to me*	Sinto que a voz é minha
	T2.Parecia que minha voz pertencia a mim	R2.*I feel my voice belongs to me*	
	VCP.Sinto que a minha voz me pertence		
2. *It seemed like my voice was functioning as it normally does*	T1.Parecia que minha voz estava funcionando normalmente	R1.*I feel that my voice functions normally*	Sinto que minha voz funciona normalmente
	T2.Parecia que minha voz estava saindo como ela normalmente sai	R2*.I feel my voice functioning normally*	
	VCP.Sinto que minha voz funciona normalmente		
*3. It seemed like my voice reflected who I am*	T1.Parecia que minha voz refletia quem eu sou	R1*.I feel that my voice reflects who I am*	Sinto que minha voz reflete quem eu sou
	T2. Parecia que minha voz refletia quem eu sou	R2*.I feel my voice reflects who I am*	
	VCP.Sinto que minha voz reflete quem eu sou		
*4. It seemed like I was in control of my voice*	T1.Parecia que eu estava no controle da minha voz	R1.*I feel that I’m in control of my own voice*	Sinto que controlo minha voz
	T2.Parecia que eu estava no controle da minha voz	R2.*I feel control over my voice*	
	VCP.Sinto que estou no controle da minha voz		
5. *I am satisfied with how my voice sounded*	T1.Estou satisfeito(a) pela forma como minha voz soou	R1.*I feel satisfied with the sound of my voice*	Fico satisfeito(a) com o som da minha voz
	T2.Estou satisfeito(a) com minha voz	R2.*I feel satisfied with my voice*	
	VCP.Fico satisfeito(a) com o som da minha voz		
6*. I was thinking about the way my voice sounded*	T1.Eu estava pensando sobre como minha voz soava	R1.*I’ve been thinking about how my voice resonates/echo*	Fico pensando sobre como minha voz soa
	T2.Eu estava pensando na maneira como minha voz saía	R2.*keep thinking about how my voice sounds*	
	VCP.Fico pensando sobre como minha voz soa		
7. *I feel as though my voice sounded clear*	T1.Eu sinto como se minha voz soasse clara	R1*.I feel that my voice is clear*	Sinto que minha voz é clara
	T2.Eu sinto como se minha voz saísse com clareza	R2*.I feel my voice clear*	
	VCP.Sinto que minha voz é clara		
8. *I feel as though I was speaking in a way that I was easily understood*	T1.Eu sinto como se estivesse falando de uma maneira que eu fosse facilmente compreendida	R1*.I feel that I talk in a way that is easily understood by others*	Sinto que minha fala é facilmente compreendida
	T2.Eu sinto como se eu estivesse falando de uma maneira que eu fosse facilmente entendido(a)	R2.*I feel I talk in a way so that I am easily understood*	
	VCP.Sinto que falo de uma forma que sou facilmente compreendido(a)		
9. *I feel as though my voice accurately reflected my mood*	T1.Sinto como se minha voz refletisse com precisão meu humor.	R1.*I feel that my voice accurately reflects my sense of humor*	Sinto que minha voz reflete como está meu humor
	T2.Eu sinto como se minha voz refletisse com precisão meu humor	R2.*I feel my voice reflects my mood in a correct way*	
	VCP.Sinto que minha voz reflete corretamente meu humor		
10*. I feel as though my voice reflected my gender*	T1.Eu sinto como se minha voz refletisse meu gênero.	R1*.I feel that my voice reflects my gender*	Sinto que minha voz reflete meu gênero
	T2.Eu sinto como se minha voz refletisse meu gênero	R2.*I feel my voice reflects my gender*	
	VCP.Sinto que minha voz reflete meu gênero		
***Transgender Congruence Scale***	**Escala de Congruência da Pessoa Transgênero**	***Congruence scale of a transgender person***	**Escala de Congruência da Pessoa Transgênero**
*0. strongly Disagree*	0.discordo totalmente	*0.I totally disagree*	0.discordo totalmente
*1. disagree*	1.discordo	*1.disagree*	1.discordo
*2. neutral*	2.neutro	*2.neutral*	2.neutro
*3. agree*	3.concordo	*3.I agree*	3.concordo
*4. strongly Agree*	4.concordo totalmente	*4.I totally agree*	4.concordo totalmente
1*. My outward appearance represents my gender identity*	T1.Meu corpo físico representa minha identidade de gênero	R1.My physical body represents my gender identity	Minha aparência representa minha identidade de gênero
	T2.Meu corpo físico representa minha identidade de gênero	R2. My physical body represents my gender identity	
	VCP. Meu corpo físico representa minha identidade de gênero		
2*. I experience a sense of unity between my gender identity and my body.*	T1. Eu sinto uma unidade entre minha identidade de gênero e meu corpo	R1. I feel that my gender and my body connected with each other	Eu sinto que existe unidade entre meu gênero e meu corpo
	T2. Eu experimento um senso de unidade entre meu gênero e meu corpo	R2. I feel there is a unity between my gender and my body	
	VCP. Eu sinto que existe uma unidade entre meu gênero e meu corpo		
3. *My physical appearance adequately expresses my gender identity*	T1.Minha aparência física expressa adequadamente minha identidade de gênero	R1.My physical appearance adequately expresses my gender identity	Minha aparência física expressa minha identidade de gênero
	T2.Minha aparência física expressa adequadamente minha identidade de gênero	R2.My physical appearance expresses in an adequate way my gender identity	
	VCP.Minha aparência física expressa adequadamente minha identidade de gênero		
4. *I am generally comfortable with how others perceive my gender identity when they look at me*	T1.Geralmente me sinto confortável com a forma como os outros percebem minha identidade de gênero quando olham para mim	R1.I generally feel comfortable with how other people notice my gender identity and look at me	Eu me sinto confortável com a forma que os outros percebem minha identidade de gênero quando olham para mim
	T2.Eu geralmente me sinto confortável com a maneira que os outros percebem minha identidade de gênero quando olham para mim	R2.Normally I feel comfortable about how others perceive my gender identity when they look at me	
	VCP.Eu geralmente me sinto confortável como os outros percebem minha identidade de gênero ao olharem para mim		
5*. My physical body represents my gender identity*	T1.Meu corpo físico representa minha identidade de gênero	R1.My physical body represents my gender identity	Meu corpo físico representa minha identidade de gênero
	T2.Meu corpo físico representa minha identidade de gênero	R2.My physical body represents my gender identity	
	VCP. Meu corpo físico representa minha identidade de gênero		
*6. The way my body currently looks does not represent my gender identity*	T1.A aparência do meu corpo atualmente não representa minha identidade de gênero	R1.The actual appearance of my physical body is not an accurate representation of my gender identity	A aparência do meu corpo não representa minha identidade de gênero
	T2.A maneira que meu corpo atualmente aparenta não representa minha identidade de gênero	R2.My actual appearance does not represent my gender identity	
	VCP.A aparência atual do meu corpo não representa minha identidade de gênero		
7*. I am happy with the way my appearance expresses my gender identity*	T1.Estou feliz com a forma como minha aparência expressa minha identidade de gênero	R1.I am happy with the way that my appearance expresses my gender identity	Eu estou feliz com a forma que minha aparência expressa minha identidade de gênero
	T2.Eu estou feliz com a maneira que minha aparência expressa minha identidade de gênero	R2. I am happy with the way my appearance expresses my gender identity	
	VCP.Eu estou feliz com a maneira que minha aparência expressa minha identidade de gênero		
8. *I do not feel that my appearance reflects my gender identity*	T1.Não sinto que minha aparência reflita minha identidade de gênero	R1.I don’t feel that my appearance reflects my gender identity	Eu estou feliz com a forma que minha aparência expressa minha identidade de gênero
	T2.Eu não sinto que minha aparência reflete minha identidade de gênero	R2.I do not feel that my appearance reflects my gender identity	
	VCP.Eu não sinto que minha aparência reflete minha identidade de gênero		
9. *I feel that my mind and body are consistent with one another*	T1.Sinto que minha mente e meu corpo são consistentes um com o outro	R1.I feel that my body and mind are coherent with one another	Eu sinto que minha mente e corpo são consistentes um com o outro
	T2.Eu sinto que minha mente e corpo são consistentes um com o outro	R2.I feel my mind and body are coherent with one another	
	VCP.Eu sinto que minha mente e corpo são coerentes um com o outro		
10. *I am not proud of my gender identity*	T1.Eu não tenho orgulho da minha identidade de gênero	R1.I don’t feel proud of my gender identity	Eu não me orgulho da minha identidade de gênero
	T2.Eu não estou orgulhoso(a) da minha identidade de gênero	R2.I do not feel proud about my gender identity	
	VCP. Eu não me orgulho da minha identidade de gênero		
11. *I am happy that I have the gender identity that I do*	T1.Estou feliz por ter a identidade de gênero que tenho	R1.I am happy with my gender identity	Eu estou feliz com minha identidade de gênero
	T2.Eu estou feliz por ter a identidade de gênero que tenho	R2.I am happy about the gender identity I possess	
	VCP.Eu estou feliz pela identidade de gênero que tenho		
12. *I have accepted my gender identity*	T1.Aceitei minha identidade de gênero	R1.I accept my gender identity	Eu aceito minha identidade de gênero
	T2.Eu aceitei minha identidade de gênero	R2.I accept my gender identity	
	VCP.Eu aceito minha identidade de gênero		

**Legenda:** T1 = tradutor 1 inglês-português; T2 = tradutor 2 inglês-português; R1 = retrotradutor 1 português-inglês; R2 = retrotradutor 2 português-inglês; VCP = versão em português do consenso entre os dois tradutores

No questionário *TSC* houve discordância quanto à forma na etapa de tradução para os itens 2, 4, 6, 7 e 10: item 2. “Eu sinto uma unidade entre minha identidade de gênero e meu corpo”/“Eu experimento um senso de unidade entre meu gênero e meu corpo”; item 4. “Geralmente me sinto confortável com a forma como os outros percebem minha identidade de gênero quando olham para mim”/“Eu geralmente me sinto confortável com a maneira que os outros percebem minha identidade de gênero quando olham para mim”; item 6. “A aparência do meu corpo atualmente não representa minha identidade de gênero”/“A maneira que meu corpo atualmente aparenta não representa minha identidade de gênero”; item 7. “Estou feliz com a forma como minha aparência expressa minha identidade de gênero”/“Eu estou feliz com a maneira que minha aparência expressa minha identidade de gênero”; item 10. “Eu não tenho orgulho da minha identidade de gênero”/“Eu não estou orgulhoso(a) da minha identidade de gênero”.

No consenso foram resolvidas as discordâncias e simplificados os itens, elaborando versões de síntese: item 2. “Eu sinto que existe uma unidade entre meu gênero e meu corpo”; item 4. “Eu geralmente me sinto confortável como os outros percebem minha identidade de gênero ao olharem para mim”, item 6. “A aparência atual do meu corpo não representa minha identidade de gênero”; item 7. “Eu estou feliz com a maneira que minha aparência expressa minha identidade de gênero”; e item 10. “Eu não me orgulho da minha identidade de gênero”.

Na etapa de retrotradução do *TSC* houve discordância quanto à forma nos itens 2, 3, 4, 6 e 11; item 2. *“I feel that my gender and my body connected with each other”/“I feel there is a unity between my gender and my body”*; item 3. *“My physical appearance adequately expresses my gender identity”/“My physical appearance expresses in an adequate way my gender identity”*; item 4. *“I generally feel comfortable with how other people notice my gender identity and look at me”/“Normally I feel comfortable about how others perceive my gender identity when they look at me”*; item 6. *“The actual appearance of my physical body is not an accurate representation of my gender identity”/“My actual appearance does not represent my gender identity”*; item 11. *“I am happy with my gender identity”/“I am happy about the gender identity I possess”*.

Na versão final após a reunião do comitê de especialistas ocorreu ajustes nos itens 1, 3, 4, 6, 7, 8 e 9 (Quadro 1).

Na etapa de pré-teste, para todos os itens da *VCS e TSC*, a opção não aplicável apresentou proporção significativamente menor que as opções da chave de resposta habitual dos instrumentos (p<0,001, para todos), conforme mostra a Tabela 1.

**Tabela 1:** Comparação da proporção de itens com respostas habituais e da proporção de itens com resposta não aplicável nos testes *Voice Congruence Scale e Transgender Scale Congruence*

Item *Voice Congruence Scale*	0-4		Não-aplicável		p-valor
	n	Proporção	n	Proporção	
		observada		observada	
*Voice Congruence Scale*					
1-Sinto que a voz é minha	37	0,97	1	0,03	0,000
2-Sinto que minha voz funciona normalmente	38	1,00	0	0,00	0,000
3-Sinto que minha voz reflete quem eu sou	38	1,00	0	0,00	0,000
4-Sinto que controlo minha voz	37	0,97	1	0,03	0,000
5-Fico satisfeito(a) com o som da minha voz	38	1,00	0	0,00	0,000
6-Fico pensando como minha voz soa	37	0,97	1	0,03	0,000
7-Sinto que minha voz é clara	37	0,97	1	0,03	0,000
8-Sinto que minha fala é facilmente compreendida	38	1,00	0	0,00	0,000
9-Sinto que minha voz reflete como está meu humor	38	1,00	0	0,00	0,000
10-Sinto que minha voz reflete meu gênero	38	1,00	0	0,00	0,000
*Transgender Scale Congruence*					
1-Minha aparência representa minha identidade de gênero	38	1,00	0	0,00	0,000
2-Eu sinto que existe unidade entre meu gênero e meu corpo	37	0,97	1	0,03	0,000
3-Minha aparência física expressa minha identidade de gênero	38	1,00	0	0,00	0,000
4-Eu me sinto confortável com a forma que os outros percebem	38	1,00	0	0,00	0,000
minha identidade de gênero quando olham para mim					
5-Meu corpo físico representa minha identidade de gênero	37	0,97	1	0,03	0,000
6-A aparência do meu corpo não representa minha identidade de	36	0,95	2	0,05	0,000
gênero					
7-Eu estou feliz com a forma que minha aparência expressa minha	38	1,00	0	0,00	0,000
identidade de gênero					
8-Eu não sinto que minha aparência reflete minha identidade de	38	1,00	0	0,00	0,000
gênero					
9-Eu sinto que minha mente e corpo são consistentes um com o	37	0,97	1	0,03	0,000
outro					
10-Eu não me orgulho da minha identidade de gênero	37	0,97	1	0,03	0,000
11-Eu estou feliz com minha identidade de gênero	36	0,95	2	0,05	0,000
12-Eu aceito minha identidade de gênero	36	0,95	2	0,05	0,000

Teste Binominal

**Legenda:** n = frequência absoluta

## DISCUSSÃO

A adaptação transcultural de um questionário é a etapa inicial do processo de validação, essencial para que esse instrumento possa ser utilizado em uma língua e cultura diferente daquela em que foi criado^(23)^.

Após as etapas de tradução, síntese dos pesquisadores, retrotradução e consenso dos especialistas foi necessária a realização de ajustes nos itens dos instrumentos para os tornarem compreensíveis à cultura brasileira. A versão original em inglês do *VCS* utiliza-se o tempo verbal no passado, em virtude de o questionário estar inserido em uma pesquisa que objetivava identificar a percepção da pessoa logo após a realização de uma tarefa de fala. Observou-se a necessidade de correção do tempo verbal, transformando-o para o presente do indicativo.

Foi necessária também a inserção na versão em português do *VCS* da frase introdutória “Quando eu me comunico com as pessoas...” antes de todos os itens, para facilitar a compreensão do respondente. Desta forma, foi possível articular os itens à comunicação.

A simplificação de itens, omissão de termos que não faziam diferença na construção da frase, e a substituição de vocábulos no *TSC* também se fizeram necessárias.

No pré-teste constatou-se que todos os itens dos dois instrumentos se aplicavam e estavam compreensíveis para a população trans. No *VCS* a opção não-aplicável foi marcada por um único indivíduo para os itens 1, 4, 6 e 7: item 1. “Sinto que a voz é minha”; item 4. “Sinto que controlo minha voz”; item 6. “Fico pensando como minha voz soa”; item 7. “Sinto que minha voz é clara”.

No *TSC* uma pessoa marcou não-aplicável para os itens 2, 5, 9 e 10: item 2. “Eu sinto que existe unidade entre meu gênero e meu corpo”; item 5. “Meu corpo físico representa minha identidade de gênero”; item 9. “Eu sinto que minha mente e corpo são consistentes um com o outro”; item 10. “Eu não me orgulho da minha identidade de gênero”. Neste mesmo questionário, dois participantes assinalaram não-aplicável para os itens 6, 11 e 12: “A aparência do meu corpo não representa minha identidade de gênero”, “Eu estou feliz com minha identidade de gênero”, “Eu aceito minha identidade de gênero”. Contudo, essa foi uma situação isolada e tais respostas não expressaram a reação geral dos respondentes ao instrumento. Portanto, novos ajustes não foram considerados necessários. A adaptação transcultural, realizada neste estudo, produziu as versões em PB de ambos os questionários, que são aplicáveis e de fácil compreensão para a pessoa trans, contribuindo para uma avaliação fonoaudiológica mais específica e adequada. As versões finais aqui apresentadas passarão pelas próximas fases do processo de validação, para confirmação do número de itens e fatores, e análise das propriedades psicométricas dos instrumentos. Com a conclusão desse processo, os instrumentos estarão prontos para uso clínico e em pesquisa com a população trans.

## CONCLUSÃO

Na adaptação transcultural para o português brasileiro dos protocolos *VCS* e *TSC* foram necessários ajustes para que os instrumentos ficassem adequados ao português brasileiro. As versões transculturalmente adaptadas dos protocolos foram denominadas Escala de Congruência Vocal e Escala de Congruência da Pessoa Transgênero. Ambos os instrumentos mostraram ser de fácil compreensão e podem ser utilizados na clínica e na pesquisa para analisar a compreensão da percepção de congruência de gênero, vocal e corporal, vivenciada pelas pessoas trans.
